# Biosynthesis of CuO nanoparticles using aqueous extract of herbal tea (*Stachys Lavandulifolia*) flowers and evaluation of its catalytic activity

**DOI:** 10.1038/s41598-021-81320-6

**Published:** 2021-01-21

**Authors:** Hojat Veisi, Bikash Karmakar, Taiebeh Tamoradi, Saba Hemmati, Malak Hekmati, Mona Hamelian

**Affiliations:** 1 Department of Chemistry, Payame Noor University, Tehran, Iran; 2 Department of Chemistry, Gobardanga Hindu College, 24-Parganas (North), India; 3 Department of Organic Chemistry, Faculty of Pharmaceutical Chemistry, Tehran Medical Sciences, Islamic Azad University, Tehran, Iran

**Keywords:** Biosynthesis, Catalysis

## Abstract

Plant derived biogenic synthesis of nanoparticles (NP) has been the recent trend in material science as featured sustainable catalysts. A great deal of the current nanocatalytic research has been oriented on the bio-inspired green catalysts based on their wide applicability. In this context, CuO NPs are synthesized following a green approach using an herbal tea *(Stachys Lavandulifolia)* flower extract. The phytochemicals contained in it were used asthe internal reductant without applying harsh chemicals or strong heat. The derived nanoparticles also got stabilized by the biomolecular capping. The as-synthesized CuO NPs was characterized over FT-IR, FE-SEM, EDS, TEM, XRD, TGA and UV–Vis spectroscopy. These NPs were exploited as a competent catalyst in the aryl and heteroaryl C–heteroatom (N, O, S) cross coupling reactions affording outstanding yields. The nanocatalyst was isolated and recycled in 8 consecutive runs with reproducible catalytic activity. Rigidity of the CuO/*S. Lavandulifolia* nanocomposite was further justified by leaching test and heterogeneity test.

## Introduction

**Figure 1:**
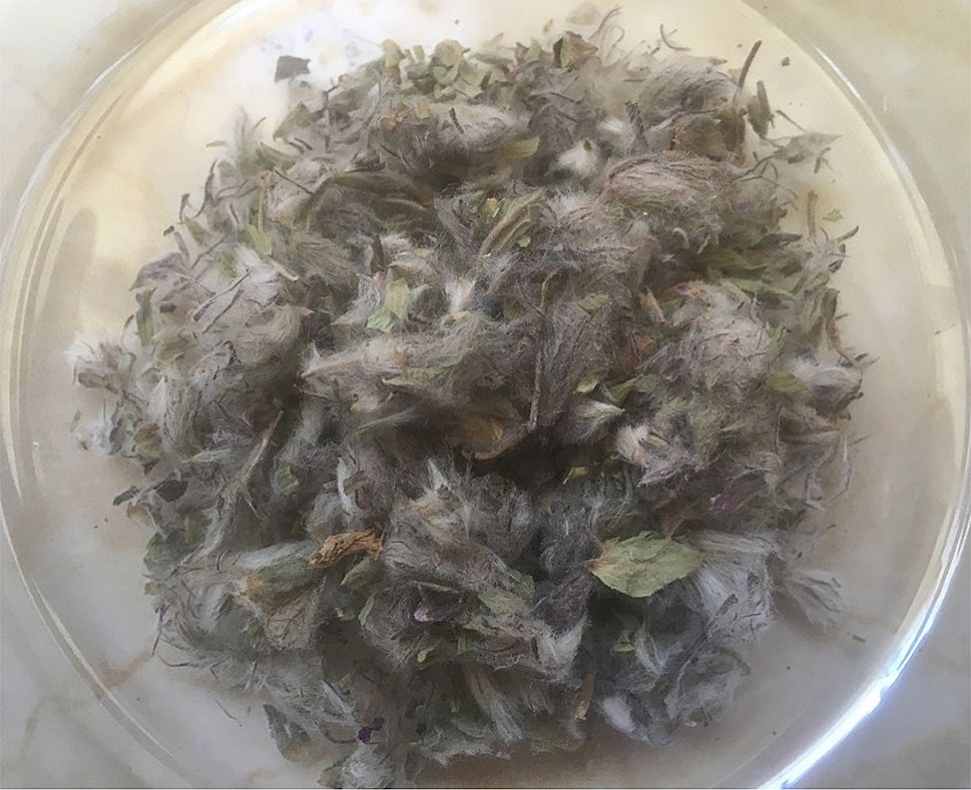
Image of *Stachys lavandulifolia* genum.

In the last few years designing of nanoparticles with tunable morphology with controlled shape, dimension and orderedness has been a significant area of concern in material science. A high degree of research has been carried out in view of their tremendous applications in diverse fields like chemo and biosensing, medicinal therapeutics, particularly as antimicrobial, antibacterial, anticancer agents, drug delivery systems, energy saving devices, optics, optoelectronics, electrochemistry andcatalysis^1–9^. The nanometric dimension, unique shape and large surface area has been beneficial in their distinctive catalytic properties. Further surface modifications over these materials by post-functionalization approach introduces unique physical properties^10–13^. A number of chemical and physical approaches are being encroached for the fabrication of NPs following top down or bottom up approach^14, 15^. Nevertheless, most of the synthetic methods involve harsh conditions like strong reducing agents, elevated temperatures, high pressure and generates hazardous by-products *in-situ* which is deterrent for green protocol^16, 17^. In some cases, the methodologies are tedious and expensive too. Thereby, the exploitation of biometric, clean, and eco-friendly procedures for the synthesis of NPs has increasing demand in view of green nanotechnology^18–23^. Notably, nature has provided a great resource in this regard and can be considered as an eco-bio-laboratory. Natural resources, especially different plant derived extracts like leaves, flowers, barks, fruit juices, fruit peels etc. are advantageous over physical and chemical methods. They are easily available, abundant, cheap, environmentally benign, and most importantly contains numerous phytochemicals (e.g., polyphenols, mild acids, alkaloids, terpenoids, flavonoids etc.) that are mild and very effective in the transformation of metal precursors towards the NPs. Moreover, the biogenic NPs are internally stabilized by the biomolecules as capping agents. They also protect the NPs from self-aggregation. In the last few years quite a number of methods have been reported on the biogenic synthesis of noble metal NP^24–31^. These very advantages have encouraged us to prefer the green biometric approach in the preparation of CuO NPs over an indigenous herbal tea flowers (*Stachys lavandulifolia*) extract (Fig. 1). The plant is grown abundantly in the hills of Kermanshah, Zagros area of western Iran and is popularly known as mountain tea (*Chay-e-Kouhi*). The herb is basically used as traditional medicinal plant in Iran^32^.

**Scheme 1:**
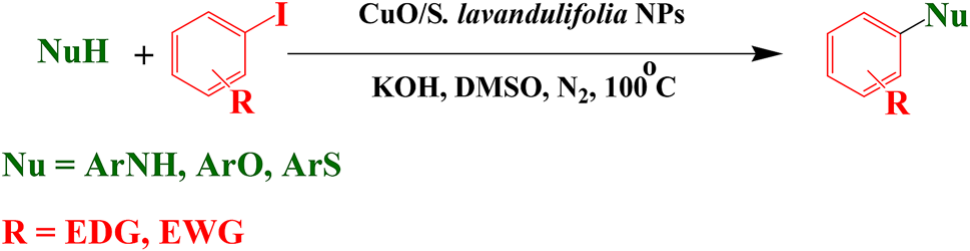
C–heteroatom cross coupling reactions over CuO NPs.

Transition metals (Pd, Pt, Au, Ag, Rh, Ir etc.) are known to be distinctive catalysts in their ability to form bonds regardless their cost and sensitivity^33–39^. In this context, Cu has its unique features in terms of its abundance, cost effectiveness, eco-benevolence and stability. The CuO NP has wide implications in the field of thermal conductance, gas sensing, magnetic recording media, solar cell applications and pharmacology^40–44^. In modern trend of research, “NP catalyzed Organic Synthesis Enhancement” (NOSE) approach has been well-admired^30, 45^. In promotion of NOSE chemistry, we herein report a green and expedient protocol for the C–heteroatom (N, O, and S) cross coupling catalyzed over biogenic CuO NP (Scheme 1)^46^. These products especially the heterocyclic coupled ones have extremely important biological, pharmaceutical and material values^47–50^.

Earlier, a number of research groups like Weingarten, Chan-Lam, Buchwald, Taillefer, Ma, Punniyamurthy have reported the Cu catalyzed carbon-heteroatom cross coupling reactions^51–58^. However, most of them used different ligand based homogeneous catalysts or heterogeneous catalysts being generated by using toxic and strong reducing agents. The reusability of catalysts and greenness has been an important issue therein. In comparison, our protocol is free from the toxic reducing agents and external stabilizers, involves low cost green procedure for the biogenesis of CuO NPs and the material being used in the efficient, ligand free synthesis of C–N/O/S coupled products at short reaction time, making the overall procedure industrially and sustainably viable.

## Experimental

### Synthesis of herbal tea extract

The fresh *S. lavandulifolia* (10 g) flowers were cleaned, dried and spread over 100 mL Milli-Q water and warmed at 60 °C for 15 min. The extract was filtered using Whatmann No. 1 filter paper and centrifuged on at 6000 rpm for 5 min to discard the probable aggregations. Finally, the clear extract was preserved at 4 ˚C for further use.

### Biogenic synthesis of CuO NPs over plant extract

In order to prepare the CuO NPs, 10 mL of the extract was mixed to 100 mL 1 mM Cu(OAc)_2_ solution and the mixture was heated at 80 °C for 100 min. The formation of NP was confirmed by change in color to dark brown, an outcome of surface plasmon resonance excitation. The resulting sediments were rinsed thrice with deionized water, chloroform and ethanol successively and finally dried in air for 48 h.

### Typical procedure for C–heteroatom cross-coupling reactions over CuO/*S. lavandulifolia* NPs

A mixture of N/O/S nucleophile (1 mmol), aryl halide (1.1 mmol), KOH (1.5 mmol) and 3 mol % CuO/*S. lavandulifolia* NPs in dry DMSO (3 mL) was heated at 100 °C at inert conditions. After completion (by TLC), the reaction was worked up by adding into water (3 mL) and the organic layer was extracted with EtOAc. It was then washed with brine solution (10 mL), dried over Na_2_SO_4_ and concentrated. The crude product was finally purified over silica gel column chromatography using EtOAc/hexane mixture as eluent.

## Results and discussion

CuO NPs were synthesized following a green synthetic approach by *Stachys lavandulifolia* herb (Fig. 1). The primary phytochemical screening of the extract showed the existence of flavonoinds, triterpenoids, steroids, cardenolides, and alkaloids. During in *situ* synthesis, these phytochemicals play as the reducing agent to convert Cu^2+^ ions to Cu NPs and simultaneously act as effective capping agent to prevent the NPs agglomerations. In the resulting procedure Cu(0) gets aerially oxidized to CuO NPs by heating. The as synthesized NPs were analytically characterized over different techniques like UV–Vis and FT-IR spectroscopy, FESEM, EDX, TEM, XRD and TGA.

**Figure 2:**
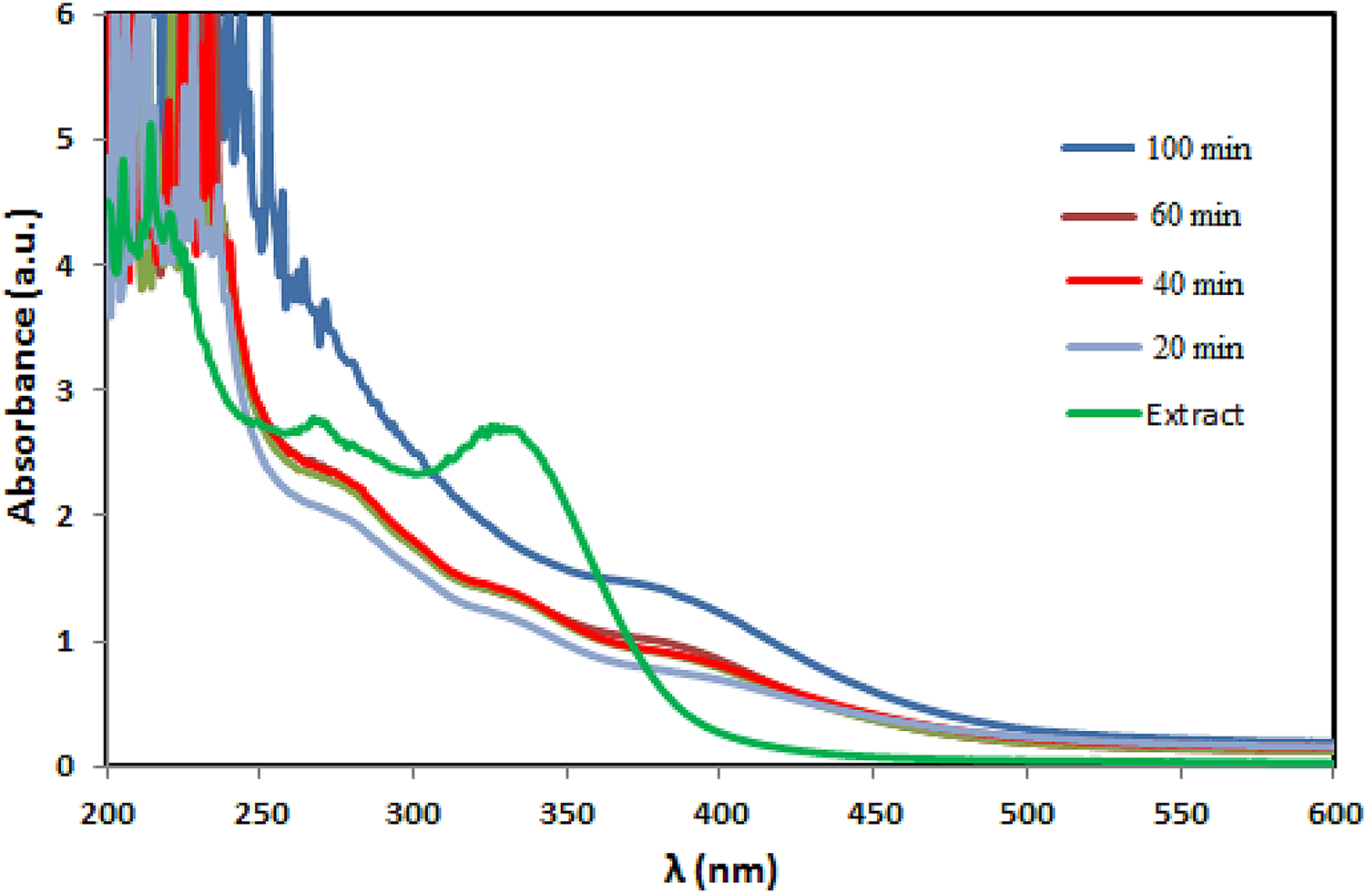
UV–Vis spectrum of biogenic CuO NPs using *S. lavandulifolia* extract.

**Figure 3:**
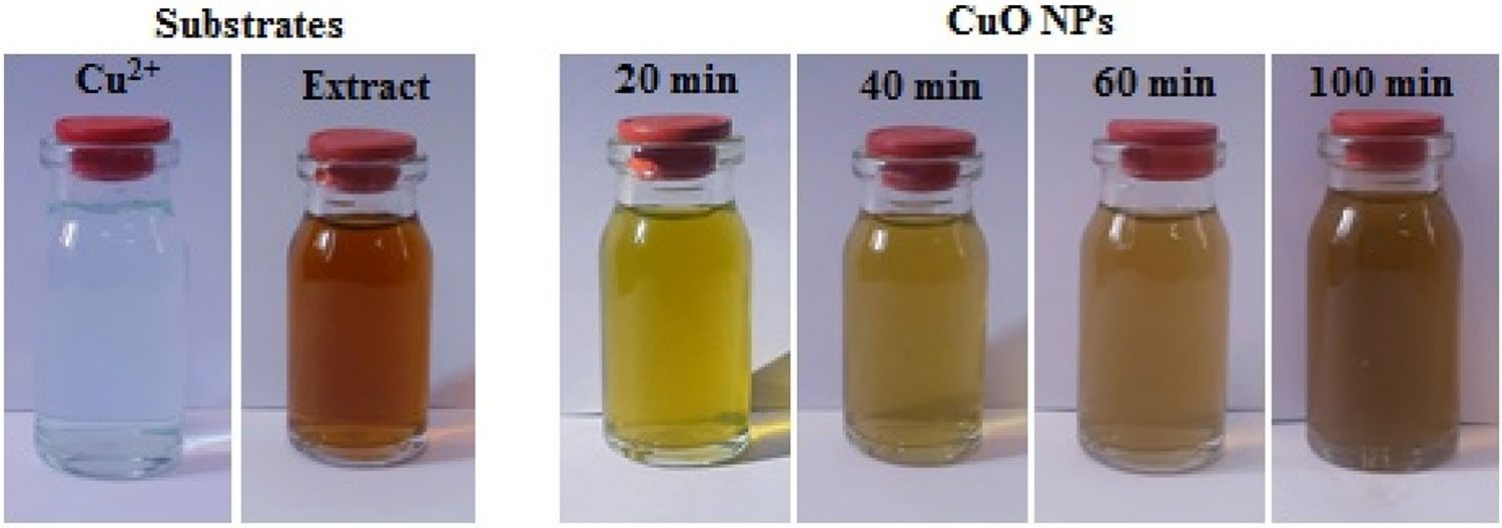
Visible detection of the biosynthesis of CuO NPs.

UV–Vis spectroscopy is a potential measure for the identification of CuO NPs. The spectrum recorded at different time intervals of the reaction are presented in Fig. 2. After 100 min, the reaction was ended with the formation of CuO NPs, which was confirmed by the characteristic surface plasmon absorbance (sky blue line) at 400 nm. The variation in color of the reaction aliquots towards the formation of CuO NPs are depicted in Fig. 3.

**Figure 4:**
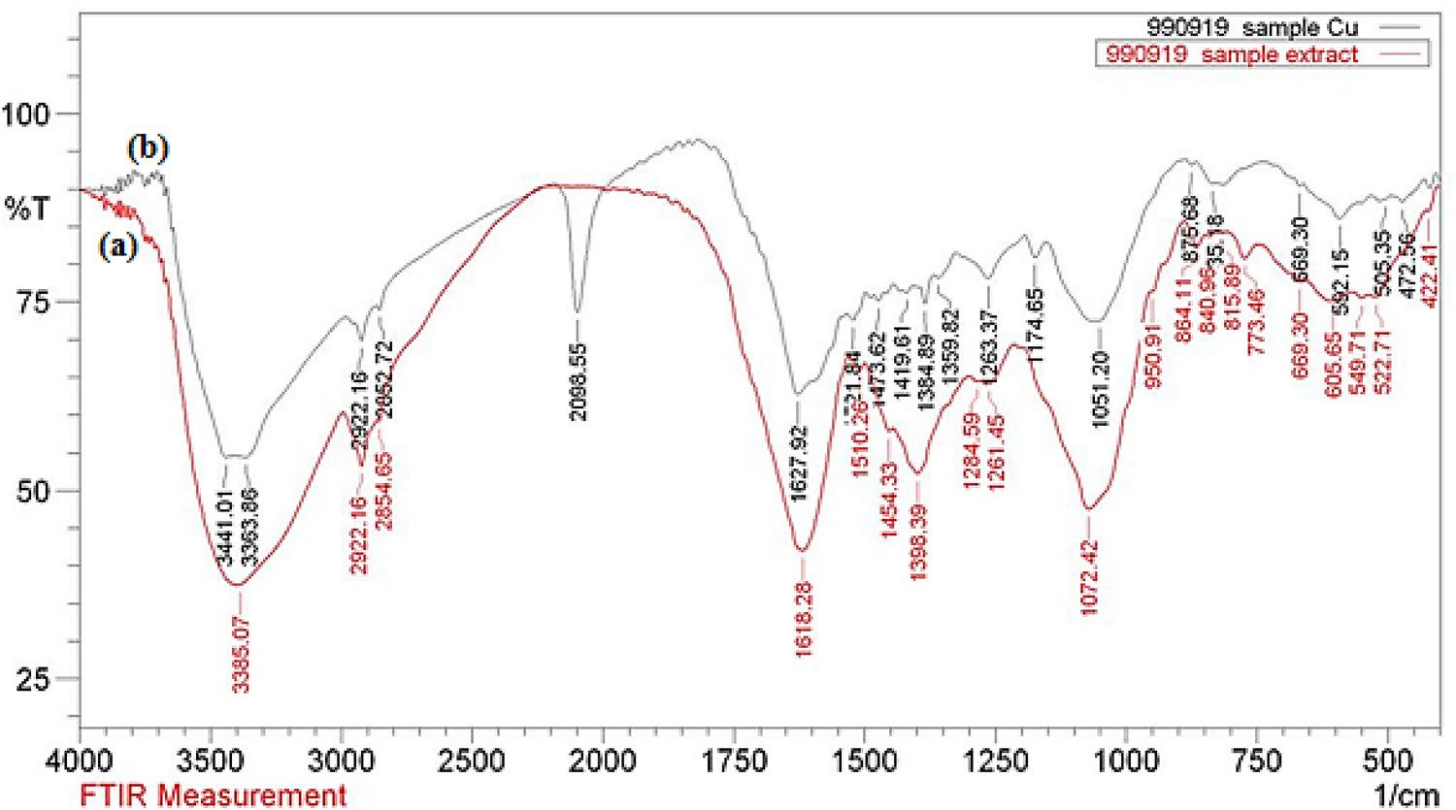
FT-IR spectrum of aqueous extract of *Stachys lavandulifolia* (**a**), and green synthesized CuO NPs (**b**).

FT-IR analysis was carried out to detect the probable biomolecules present in the aqueous extract of *S. lavandulifolia* responsible for the green synthesis of CuO NPs. Figure 4 shows the FT-IR spectrum of the plant extract and CuO/*S. lavandulifolia* nanocomposite. The spectrum of the plant extract represents several bands ranging from 3100 to 3385 cm^−1^ which are attributed to free hydroxyl groups and their intra/intermolecular H-bonds of polyphenolic compounds^59, 60^. The sharp peaks appeared at 2922, 1618 and 1398 cm^−1^ were related to saturated hydrocarbons (C_Sp3_-H) and C=O and C=C aromatic stretching frequencies respectively. The spectrum corresponding to CuO NPs is presented in Fig. 4b. The observed bands are due to lattice vibrational modes representing the functional groups of biomolecules supported NPs. The broad band around 3400 cm^−1^ was related to OH stretching frequency. The peak at 1473 cm^−1^ and 1627 cm^−1^ was assigned to the bending vibrations of sp^2^-bonds of aromatic moieties and carbonyl stretching frequencies respectively. Different organic functional moieties like amides, ethers and other aliphatic groups surrounded over CuO NP are specified by the peaks observed at 1263, 1174 and 1051 cm^−1^. The phonon bands seen at 505 and ~ 592 cm^−1^ represents the stretching vibration of Cu–O bond in monoclinic CuO^24^.

**Figure 5:**
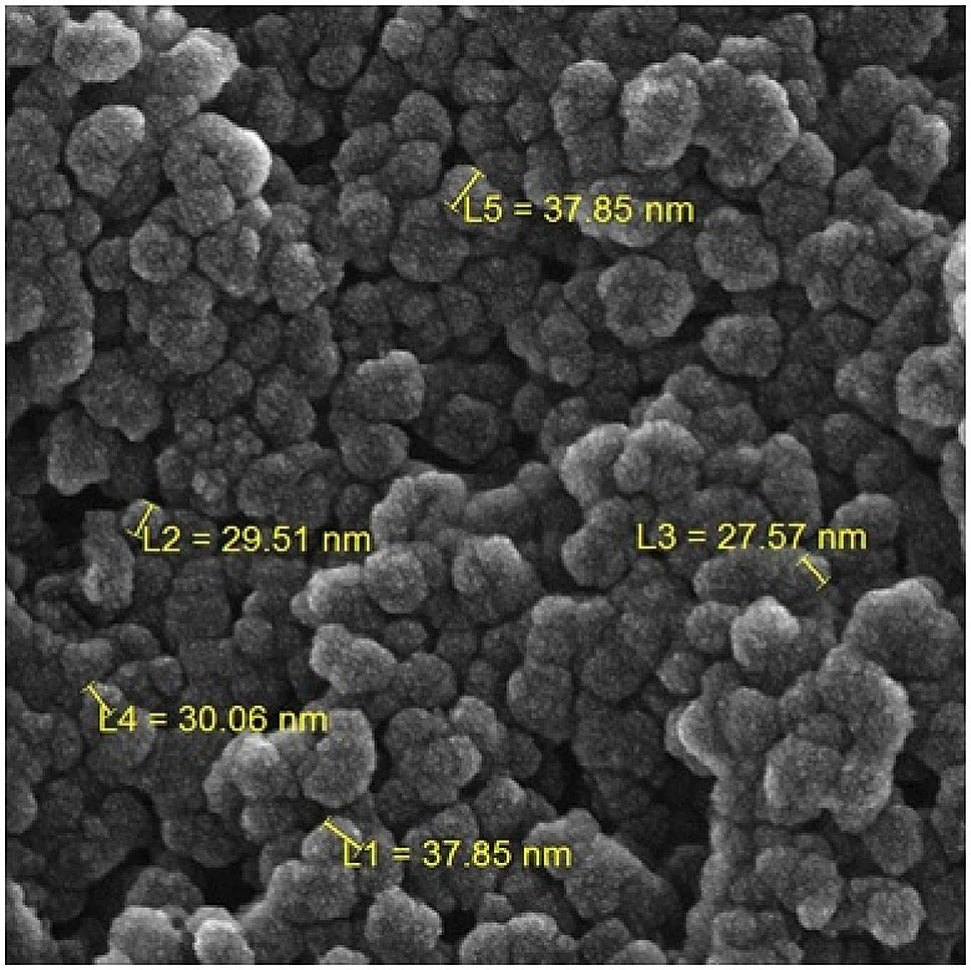
FE-SEM study of biogenic CuO NPs.

**Figure 6:**
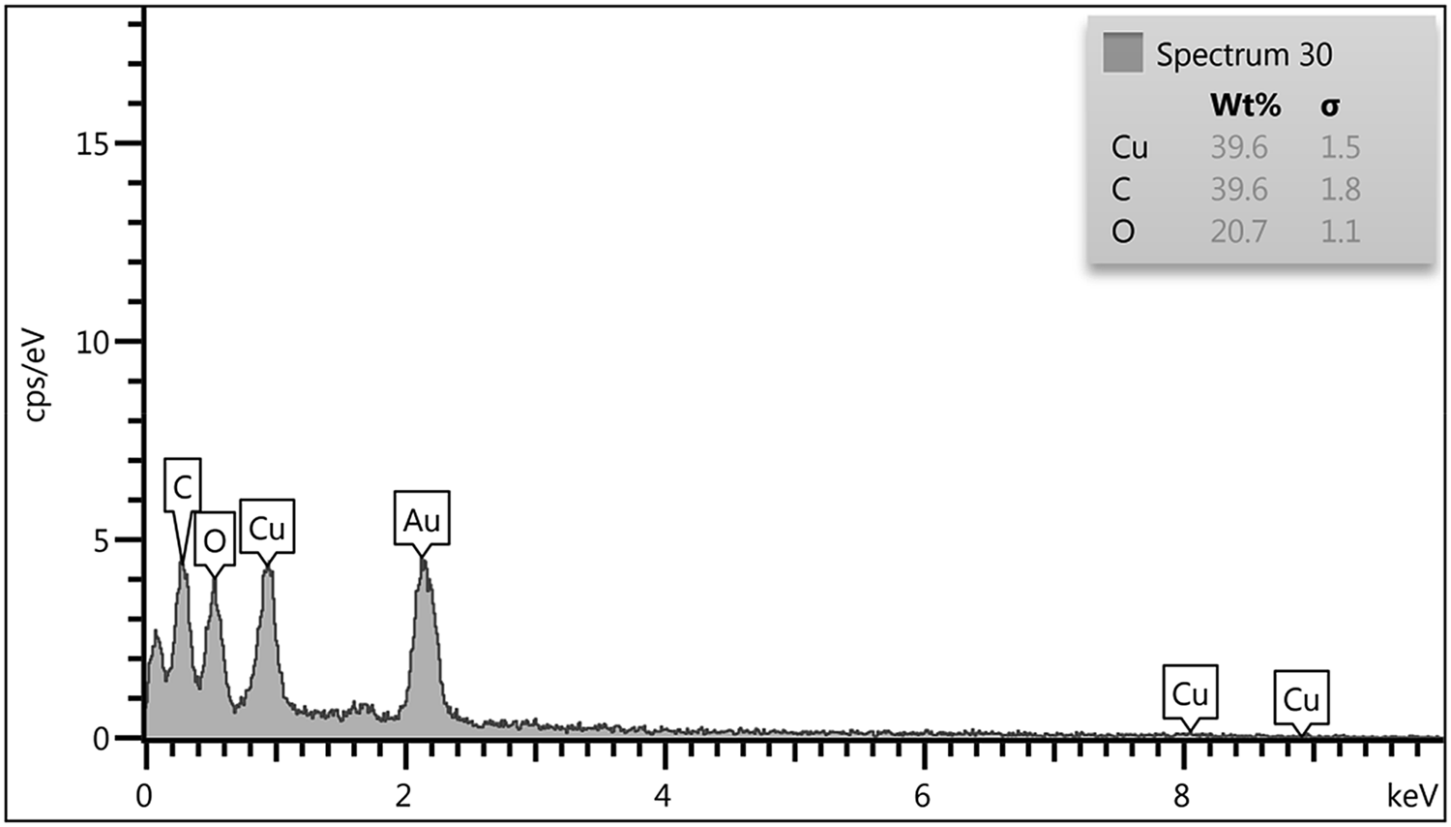
EDX analysis of biogenic CuO NPs.

The FE-SEM image presented in Fig. 5 indicates the defined spherical morphology of the prepared CuO NPs. All CuO NPs have the mean particles size of 20–35 nm. It is observed that biogenic synthesis of CuO NPs results comparably small spherical particles with uniform dimension. Clearly, small nuclear particles are self-aggregated and orient themselves to form larger spheres. Composition of the as-synthesized material being analyzed through EDX study, is observed in Fig. 6. The spectrum shows C, N, Cu and O peaks. The presence of oxygen in the profile demonstrates the oxidation of prepared Cu^0^ NPs occurred upon exposure to air. The C and N atoms correspond to the phytochemicals from plant extract being bonded to CuO NPs.

**Figure 7:**
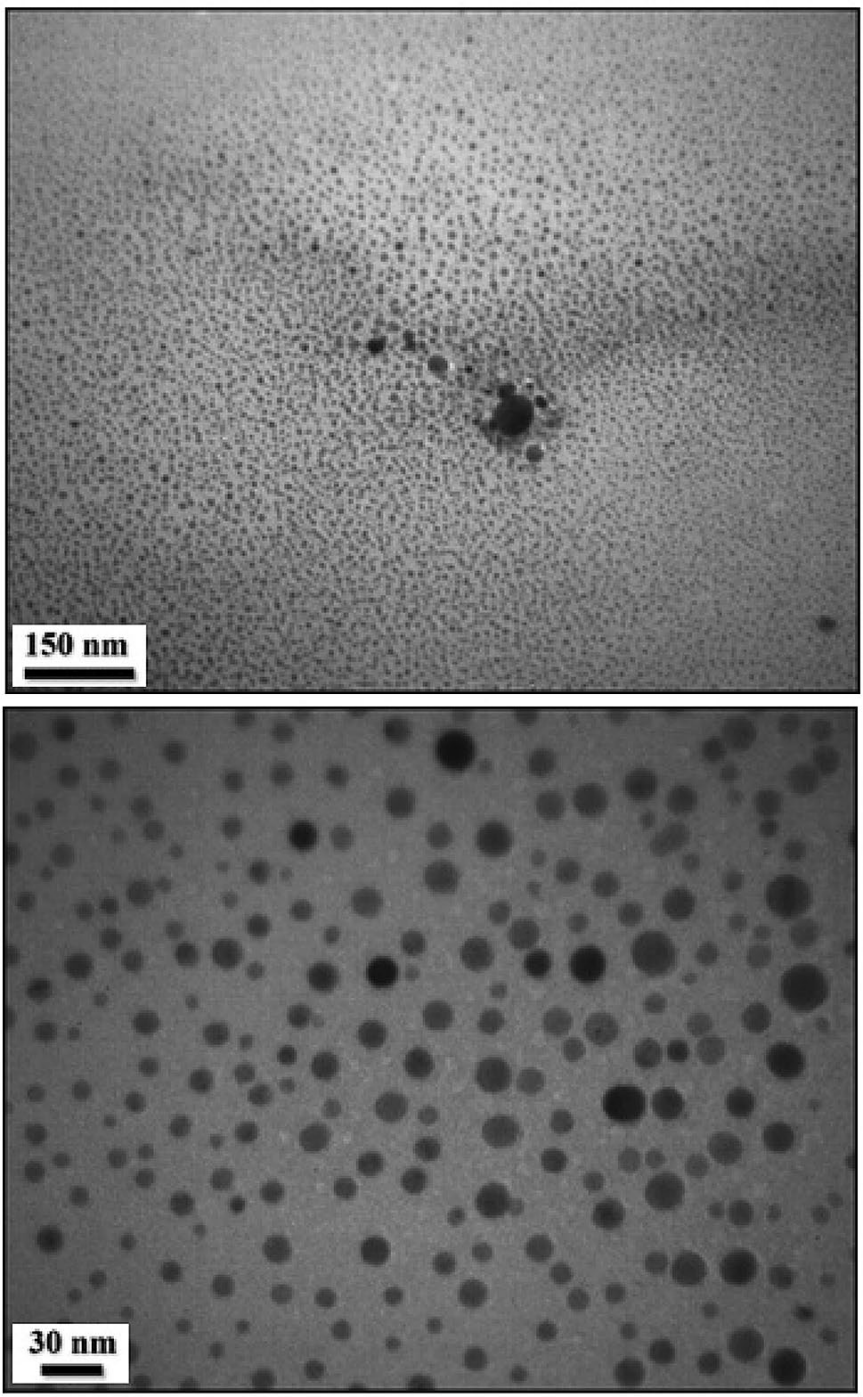
TEM images of biogenic CuO NPs.

The TEM analysis provides detailed morphological insights regarding the shape and dimension of CuO NPs. Figure 7 exhibits that the NPs are synthesized with moderately good monodispersity, having almost spherical structures ranging from 15 to 25 nm without any agglomeration.

**Figure 8:**
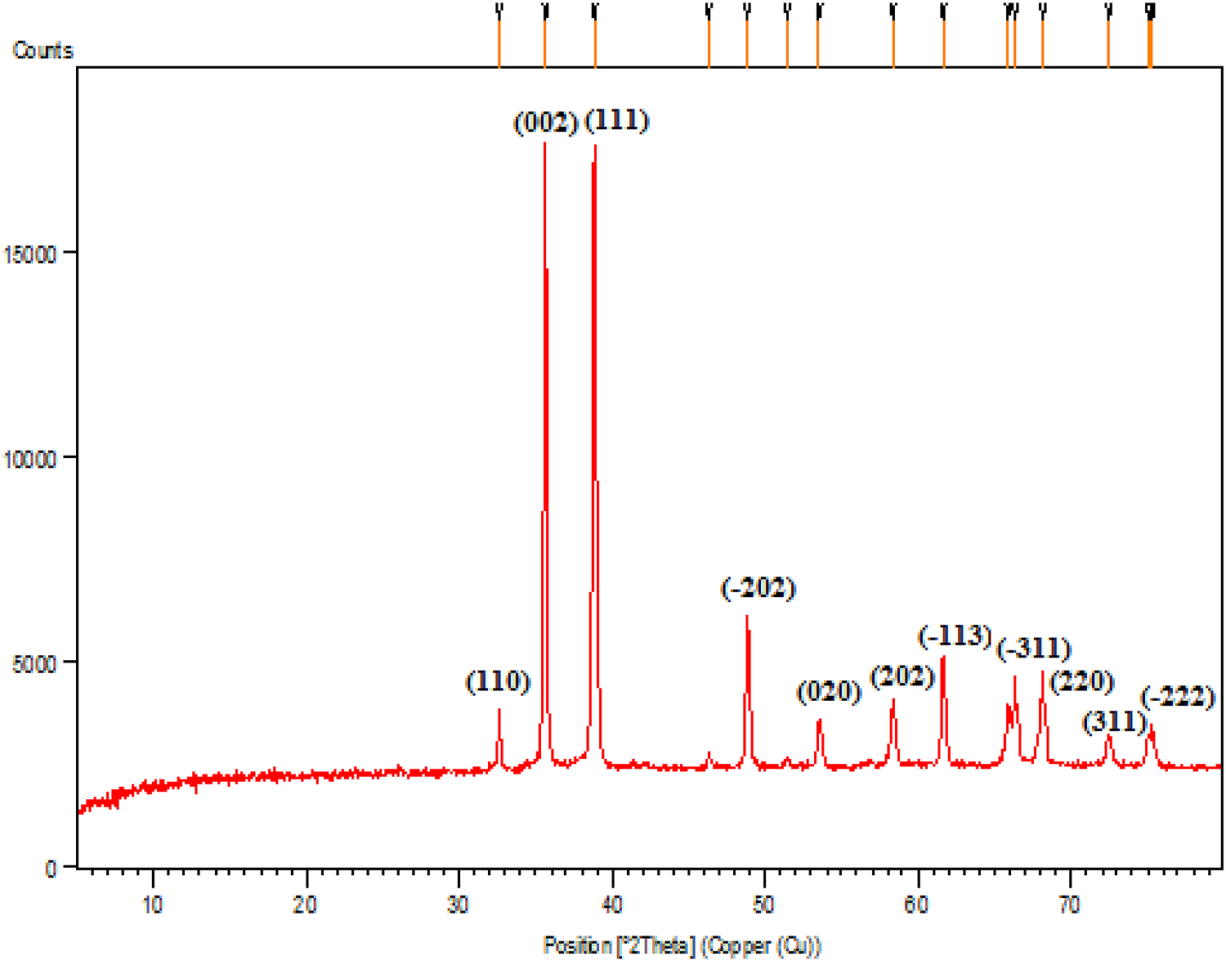
Powder XRD pattern of biogenic CuO NPs.

Crystallinity of the green prepared CuO NPs was ascertained by XRD study (Fig. 8). The observed diffraction sharp peaks position at 2θ = (32.59°, 35.61°, 38.78°, 48.82°, 53.54°, 58.37°, 61.60°, 66.31°, 68.15°, 72.46° and 75.30° were assigned to (1 1 0), (− 1 1 1), (1 1 1), (− 2 0 2), (0 2 0), (2 0 2), (− 1 1 3), (− 3 11), (2 2 0), (3 1 1) and (− 2 2 2) are highly consistent with JCPDS standard no. 01-080-0076 of CuO NPs with a monoclinic phase^61^.

**Figure 9:**
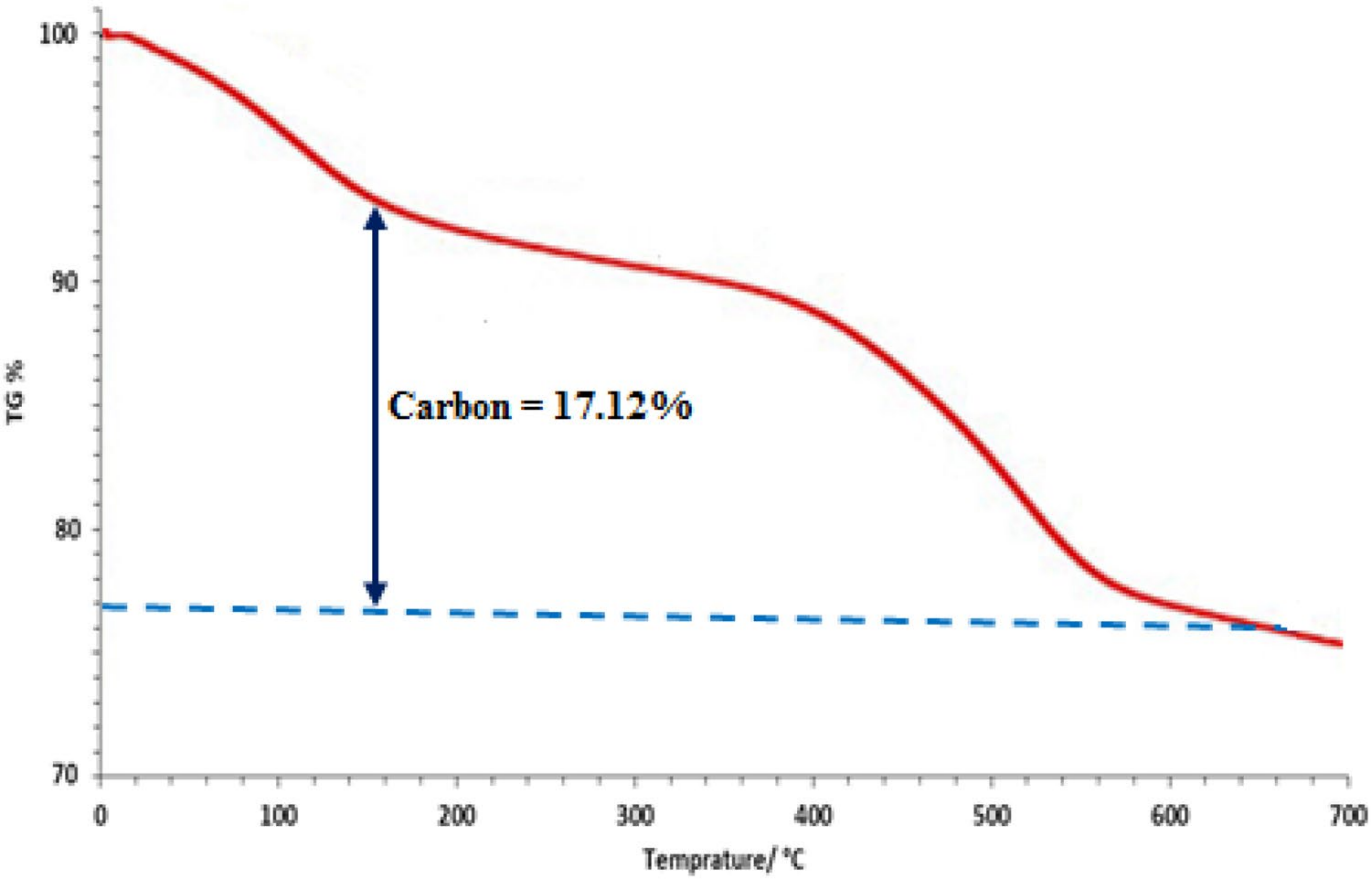
TGA profile of CuO NPs.

The level of % purity of CuO NPs in the composite was approximately 76.1 wt% as confirmed from the TGA, presented in Fig. 9. The mass decay observed in the region of < 200 °C was due to physisorbed water and organic polyphenolic bodies. Almost 17.12 wt % decrease in weight was reported at 200–600 °C resulting from the degradation of organic species. As the decomposition starts after 130 °C, the catalyst remains active at our reaction condition (100 °C) without any deformations.

**Scheme 2:**
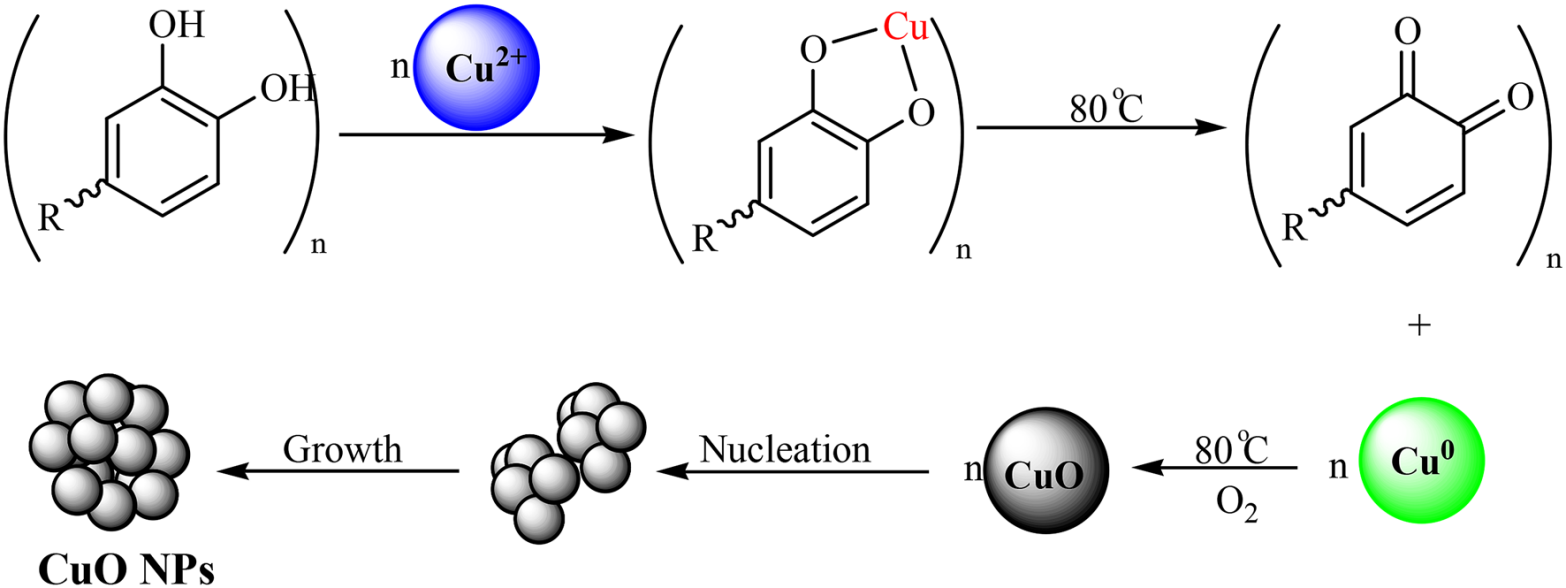
Mechanistic study for the biogenesis of CuO NPs over *Stachys lavandulifolia* extract.

There have been several reports for the bio-inspired synthesis of nanomaterials where polyphenolic compounds function as green reductants^62^. Incidentally, *Stachys lavandulifolia* is known to have a rich source of polyphenolic and flavonoidic compounds^63, 64^. These phytochemicals play the role of reductant to Cu NPs. Scheme 2 represents a possible pathway towards the biogenenis of CuO NPs. Initially, the plant extract combined with the metal salt solution results in a polyphenolic complex with the Cu^2+^ ion and consummated reduction generates Cu^0^ NPs. The metallic Cu atoms further get oxidized over atmospheric oxygen forming stable CuO NP. Nucleation of the synthesized CuO NP promotes the growth towards isolable materials.

Once having the structural affirmation of biogenic CuO NPs by rigorous instrumentation, the catalytic performance of these NPs was assessed in the carbon-heteroatom cross coupling reactions. However, prior to general studies, optimization of the reaction conditions seemed quite significant and thereby a model reaction of iodobenzene with three N, O and S nucleophiles, viz., indole, phenol and thiophenol was screened with an array of experiments applying diverse conditions like catalyst load, solvent, base and temperature. The results have been documented in Table 1. While carrying out the C–N coupling reactions of indole, it failed in the absence of catalyst and the additive base (Table 1, entry 8, 10) indicating their importance. Among the different bases used, KOH was found to be most effective resulting highest yield at 100 °C (Table 1, entry 8). We also screened the reaction in different solvents like EtOH, toluene, DMF, DMSO, CH_2_Cl_2_, CH_3_CN (entry 1–8) when the best result was achieved in DMSO in shortest interval. Again, 3 mol% catalyst load was found optimum to have the best catalytic results (Table 1, entry 8). We examined the reaction at different temperature too. However, at lower temperature conditions it was not much successful and we continued the reactions at 100 °C (Table 1, entry 15, 16). After the fruitful experimentation for the best reaction conditions with N nucleophile (indole), we continued the same with other nucleophiles like O (phenol) and S (thiophenol) and delighted to have excellent outcomes at shorter reaction times (Table 1, entry 17, 18). Therefore, the best results for the Ar–C–N/O/S coupling were obtained by heating a mixture of 1.0 mmol iodobenzene, 1.1 mmol nucleophile and 1.5 mmol KOH in DMSO over 3 mol% CuO*/S. lavandulifolia* NP as catalyst.

**Table 1:** Optimization of reaction condition for CuO/*S. lavandulifolia* NPs -catalyzed coupling of indole/phenol/benzenethiol with iodobenzene.


Entry	NuH	Cat. (mol%)	Base	Solvent	t (h)	Yield (%)^b^
1	Indole	1	KOH	EtOH	12	60
2	Indole	1	KOH	DMSO	10	78^c^
3	Indole	1	KOH	Toluene	12	70
4	Indole	1	KOH	DMF	12	70
5	Indole	1	KOH	CH_2_Cl_2_	24	45
6	Indole	1	KOH	CH_3_CN	24	55
7	Indole	2	KOH	DMSO	10	88^c^
8	Indole	3	KOH	DMSO	10	95^c^
9	Indole	3	-	DMSO	24	Trace^c^
10	Indole	-	KOH	DMSO	24	0^c^
11	Indole	3	K_2_CO_3_	DMSO	10	70^c^
12	Indole	3	Na_2_CO_3_	DMSO	10	60^c^
13	Indole	3	Et_3_N	DMSO	10	80^c^
14	Indole	3	NaHCO_3_	DMSO	10	65^c^
15	Indole	3	KOH	DMSO	10	60^d^
16	Indole	3	KOH	DMSO	10	35^e^
17	Phenol	3	KOH	DMSO	8	92f.
18	Benzenethiol	3	KOH	DMSO	7	96^ g^

Reaction conditions: N/O/S nucleophile (1.0 mmol), iodobenzene (1.1 mmol), CuO/*S. lavandulifolia* NPs, base (1.5 mmol), solvent (3.0 mL),N_2_ atmosphere; ^b^Isolated yield; ^c^100 °C; ^d^70 °C; ^e^25 °C.

After having the stabilized conditions, it was the turn to prove the generality of them over a wide range of heteroatom nucleophiles reacting with diverse aryl iodides to furnish a library of cross coupled products. The results have been shown in Table 2. Different aryl and heteroaryl N-nucleophiles like indole, imidazole and aniline reacts with high compatibility with diverse aryl iodides having electron accepting (NO_2_) and electron releasing functional groups (Cl, CH_3_, OCH_3_), affording outstanding yields (Table 2, entry 1–15). Notably, aniline reacted with the aryl iodides at a faster rate than indole or imidazoles (Table 2, entry 7–12). The same reaction trend is followed with O-nucleophiles like substituted phenol derivatives and S-nucleophiles like substituted thiophenol derivatives with different aryl iodides. Interestingly, here also the corresponding nucleophiles reacted excellently to manage very good yields irrespective of the substitutions. All the coupled products were authenticated by literature studies and also corroborated by spectroscopic analysis.

**Table 2:** CuO/*S. lavandulifolia* NPs -catalyzed coupling of amines, phenols, and thiols with aryl iodides.

Entry	Substrate	Aryl iodide	Time (h)	Yield (%)^b^	Ref^c^	TOF (h^−1^)^d^
1	Indole	C_6_H_5_I	10	95	^65^	3.16
2	Indole	*p*-CH_3_C_6_H_4_I	11	90	^76^	2.72
3	Indole	*p*-ClC_6_H_4_I	10	95	^65^	3.16
4	Indole	*p*-CH_3_OC_6_H_4_I	12	92	^65^	2.55
5	Indole	*p*-NO_2_C_6_H_4_I	9	96	^65^	3.55
6	Indole	*o*-CH_3_OC_6_H_4_I	12	90	^65^	2.50
7	Aniline	C_6_H_5_I	4	96	^66^	8.00
8	Aniline	*p*-CH_3_C_6_H_4_I	4	95	^67^	7.91
9	Aniline	*p*-ClC_6_H_4_I	4	90	^67^	7.5
10	Aniline	*p*-CH_3_OC_6_H_4_I	5	90	^70^	6.00
11	Aniline	*p*-NO_2_C_6_H_4_I	3	96	^66^	10.00
12	Aniline	*o*-CH_3_OC_6_H_4_I	2	88	^71^	14.66
13	Imidazole	C_6_H_5_I	12	90	^67^	2.50
14	Imidazole	*p*-CH_3_C_6_H_4_I	12	85	^74^	2.36
15	Imidazole	*p*-CH_3_OC_6_H_4_I	10	75	^75^	2.50
16	Phenol	C_6_H_5_I	8	92	^77^	3.83
17	Phenol	*p*-CH_3_C_6_H_4_I	9	90	^69^	3.33
18	Phenol	*p*-ClC_6_H_4_I	8	90	^71^	3.75
19	Phenol	*p*-CH_3_OC_6_H_4_I	9	92	^71^	3.40
20	Phenol	*p*-NO_2_C_6_H_4_I	7	96	^71^	4.57
21	Phenol	*o*-CH_3_OC_6_H_4_I	12	80	^68^	2.22
22	4-Methylphenol	C_6_H_5_I	12	85	^73^	2.36
23	4-Methoxylphenol	C_6_H_5_I	10	96	^68^	3.20
24	2-Methylphenol	C_6_H_5_I	15	90	^68^	2.00
25	Benzenethiol	C_6_H_5_I	7	96	^71^	4.57
26	Benzenethiol	*p*-CH_3_C_6_H_4_I	8	90	^71^	3.75
27	Benzenethiol	*p*-ClC_6_H_4_I	8	95	^71^	3.95
28	Benzenethiol	*p*-CH_3_OC_6_H_4_I	9	90	^71^	3.33
29	Benzenethiol	*p*-NO_2_C_6_H_4_I	6	98	^71^	5.44
30	Benzenethiol	*o*-CH_3_OC_6_H_4_I	10	85	^71^	2.83
31	4-Methylbenzenethiol	C_6_H_5_I	9	95	^72^	3.51
32	4-Methoxylbenzenethiol	C_6_H_5_I	6	98	^72^	5.44

Reaction conditions: amines/phenols/thiols (1.0 mmol), iodobenzene (1.1 mmol), catalyst (3 mol%), KOH (1.5 mmol), stirring in DMSO (3.0 mL) in N_2_ at 100 °C; ^b^Isolated yield; ^c^Known product; ^d^TOF, turnover frequencies (TOF = (Yield/Time)/Amount of catalyst (mol).

While concerning about the mechanism, it is anticipated that the reaction pathway involves oxidative addition and subsequently reductive elimination. At the outset, Aryl halide molecule binds to the surface of biogenic CuO NPs. At intermediate stage, the nucleophile reacts with KOH to generate the potassio salt of them which further substitutes the halide ion bonded to catalyst surface. The Aryl group and nucleophile finally adds up over the catalyst and gets eliminated as coupled product leaving behind the active catalyst.

### Study of reusability and heterogeneity

**Figure 10:**
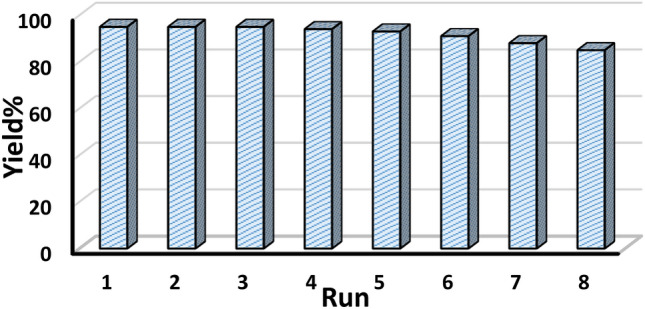
Reusable nature of the catalyst.

**Figure 11:**
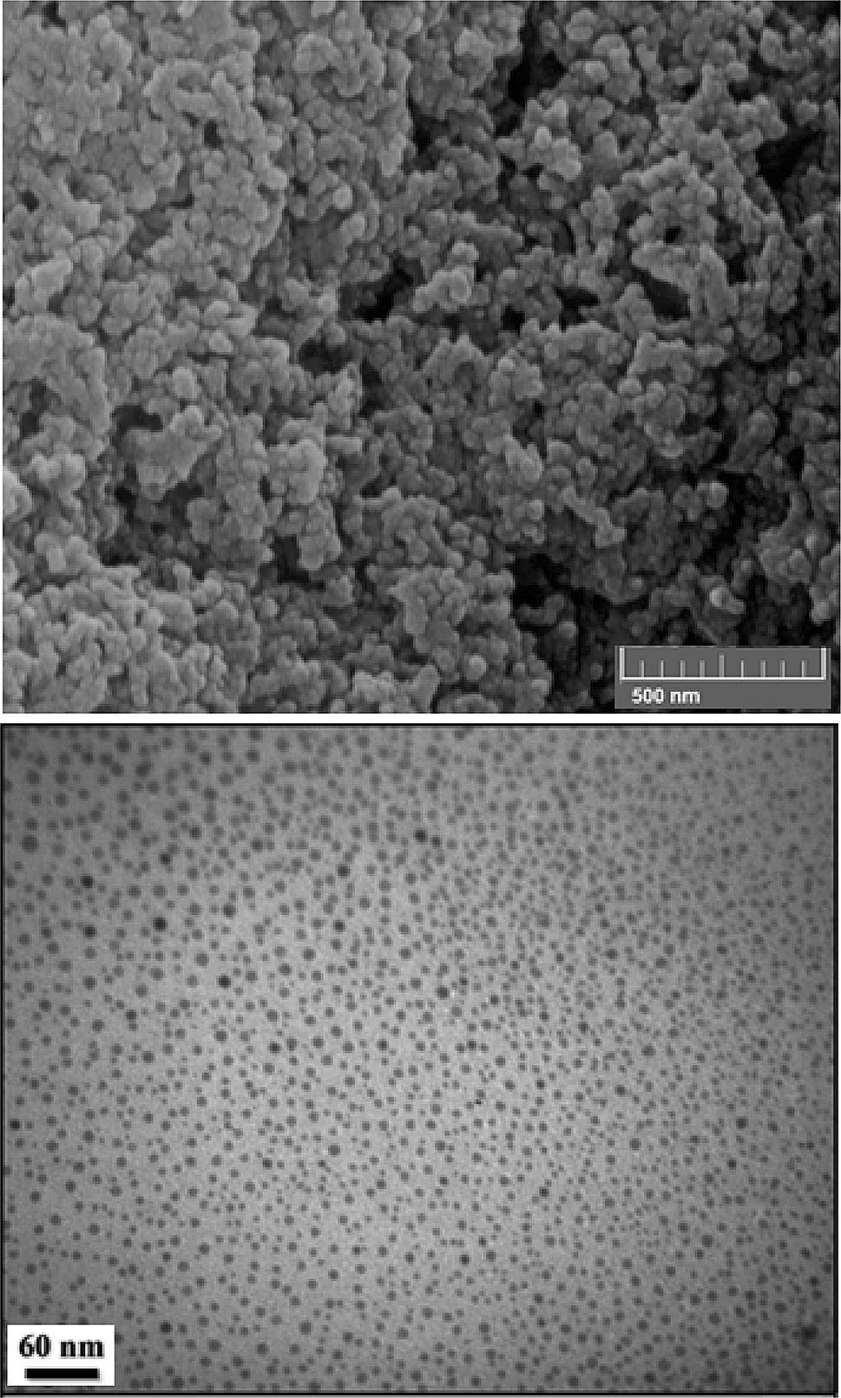
FESEM and TEM images of 7 time reused catalyst.

While concerning about the green reaction protocol, reusability of catalyst is an imperative aspect. In order of that, a probe reaction (entry 1, Table 2) with twice batch size was carried out under optimized conditions. After completion, the catalyst was retrieved on centrifuger, washed with EtOH, dried and recycled. Interestingly, we could use it for 8 successive runs without discernible change in its activity (Fig. 10). The morphology of the isolated catalyst was checked after 7th run of the reaction and found to remain intact as confirmed from FESEM and TEM analysis (Fig. 11). The catalyst was robust enough and no leaching was detected even after 7th run, as determined by ICP-OES. Furthermore, a hot filtration test was carried out to determine the heterogeneity of the catalyst, for the same above reaction. The reaction was paused after 5 h of fresh run and the catalyst was separated out of the reaction and continued further. Significantly, no additional increment in yield was detected, justifying true heterogeneity.

## Conclusion

In summary, we illustrate a bio-inspired green synthesis of CuO NPs followed by its catalytic applications. An herbal tea, *Stachys lavandulifolia* extract has been used as the plant source for biogenesis. The phytochemicals contained in *Stachys* act as bio-reductant of Cu^2+^ ions and also a stabilizer of Cu^0^ NP. Under heating conditions in air the Cu^0^ NP further gets oxidized to furnish biomolecule fabricated CuO NP. The as synthesized CuO nanocatalyst was thoroughly characterized over a wide range of physicochemical techniques. Towards its catalytic applications, the CuO/*S. lavandulifolia* nanocomposite proved its outstanding efficiency in the C–heteroatom coupling reactions of aryl and heteroaryl nucleophiles with substituted aryl iodides. The approach is effectively deployed towards diverse indole, imidazole, aniline, phenol and thiophenol derivatives in excellent yields. Hitherto known, this is the first report representative of biosynthesized CuO/*Stachys lavandulifolia* NP catalyzed C–heteroatom coupling reaction. The green protocol draws attention in terms of its simple, handy and cost-effective biogenesis of nanocatalyst, convenient operations, recyclability of catalyst and exceptional productivity.
